# “Single-use peripheral” vs “conventional” reaming in total hip arthroplasty: how to respect native centre of rotation and acetabular offset? A CT study

**DOI:** 10.1007/s00264-023-05899-3

**Published:** 2023-08-05

**Authors:** Edoardo Viglietta, Leonardo Previ, Veronica Giuliani, Giulia Rescigno, Yuri Gugliotta, Andrea Redler, Raffaele Iorio

**Affiliations:** Orthopaedic Unit, S. Andrea Hospital, University of Rome, “La Sapienza” Via Di Grottarossa 1035, Rome, Italy

**Keywords:** Total hip arthroplasty, COR, CT study

## Abstract

**Purpose:**

The respect of native hip offset represents a mainstay for satisfying results in total hip arthroplasty (THA). Historically, a great interest has been focused on restoration of femoral offset, while only in recent years, acetabular offset (AO) has been considered. The purpose of the current study was to compare the “single-use peripheral” reaming technique with the “conventional” one for the maintenance of the native COR of the hip and AO in patients undergoing to primary THA.

**Methods:**

Eighty patients affected from primary hip osteoarthritis were prospectively enrolled in the study and were divided in two groups (Group A “single-use peripheral” and Group B “conventional” reaming technique). Pre- and post-operatively, AO, acetabular floor distance (AFd) and acetabular version (AV) were assessed through a CT scan. A comparison between groups for the radiological parameters, surgical time and complications was performed.

**Results:**

The demographic data were similar in both groups. The complications rate and the AV did not differ statistically between groups. Group A presented a statistically significant shorter surgical time and lower variation between pre- and post-operative AO and AFd. Statistical significance was defined as *p* < 0.05.

**Conclusions:**

The “single-use peripheral” reaming technique demonstrated to be more reliable in reproducing the native COR and AO of patients undergoing to primary THA than the “conventional” one. The operative time was significantly reduced, and it may lead to a reduction in the infection risk even though it was not observed in the current study. Further research could be useful to validate such findings and to assess clinical impact and long-term survival of the implant.

## Introduction

The respect of native hip offset represents a mainstay for satisfying results in total hip arthroplasty (THA), providing proper function of the abductor muscles and implant stability [1–5]. Global hip offset results from the combination of both femoral offset (FO) and acetabular offset (AO). Historically, a great interest has been focused on restoration of FO, while only in the latest years AO has been considered by scientific research and industries [6–9].

AO is defined as the distance between the acetabular floor (i.e., the inner wall of the quadrilateral plate) and the centre of the femoral head [2, 8, 10–12].

**Fig. 1:**
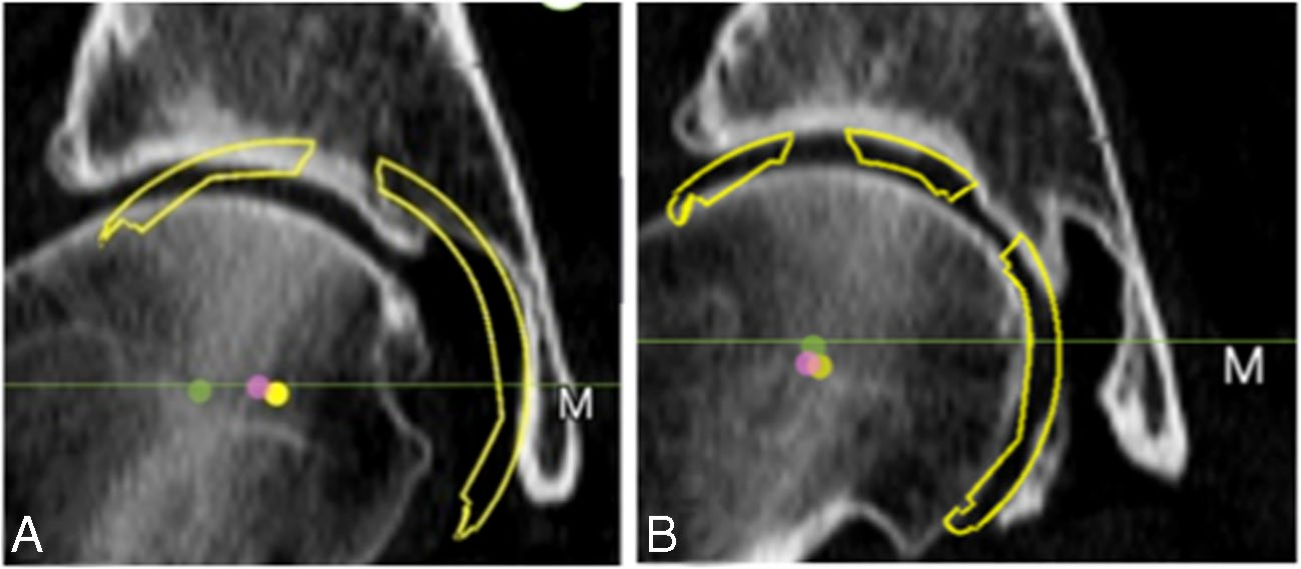
Conventional reaming (**A**) and peripheral reaming (**B**). Note the COR medialisation with the conventional reaming

During acetabular preparation in THA, conventional acetabular reaming begins with small reamer medially directed to the floor and is followed by progressively larger reamers in the desired position and until the appropriate size of the acetabular component [13]. This conventional technique has been shown to reduce the AO and displace the centre of rotation (COR) of the hip. In contrast to that, a more anatomical reaming technique which reams the acetabulum peripherally, beginning at about the same size of the femoral head without exposing the cancellous bone in the acetabular floor, has been suggested by some authors [6, 8, 14–16] (Fig. 1). This technique is supposed to maintain the AO and the COR of the hip, improving hip ROM and abductor force, preserving acetabular bone stock and reducing the risk of bony impingement and dislocation [1, 16–23]. This technique could be promising since it has been suggested not to display the COR more than 3 mm superiorly and 5 mm medially in order to obtain proper hip function and longevity [10].

The purpose of the current study was to compare the “single-use peripheral” reaming technique with the “conventional” one in the capacity to reproduce the native COR of the hip and AO in patients undergoing primary THA. The hypothesis was that the “single-use peripheral” reaming technique could more reliably reproduce the native centre of rotation of the hip and AO respect to the “conventional” technique.

## Material and methods

Between October 2020 and March 2021, 80 patients aged between 55 and 75 years with a body mass index (BMI) < 35 and affected from primary hip osteoarthritis were prospectively enrolled in the current study. Patients were randomized into two groups through computer-generated randomization numbers. Group A included 40 patients affected from hip osteoarthritis and undergoing primary THA through an anatomic acetabular reaming technique and a single-use sterile instrumentation (Groupe Lépine™, Genay, France). Group B, as control group, included 40 patients affected from hip osteoarthritis and undergoing primary THA through a conventional acetabular reaming technique. All the patients were pre-operatively informed about advantages and disadvantages of both the techniques.

Exclusion criteria consisted of the following: (1) previous surgery on the affected hip or diagnosis of secondary hip osteoarthritis; (2) severe osteophytes of the acetabular floor or severe deformity altering the normal anatomy and not allowing to reliably measure radiological parameters; (3) congenital or development diseases of the hip; (4) diagnosis of inflammatory arthropathy, autoimmune disease or rare bone disorders; (5) dementia or unwillingness to be enrolled in the study.

All the patients pre- and post-operatively received a CT scan of hip and pelvis. The hip was scanned from the antero-inferior iliac spine down to the lesser trochanter every 2 mm. All CTs were performed using the same protocol with a multibar scanner (General Electric HealthCare, 128 SLAIS, CT Scanner). The study was carried out using the Centricity™ Universal Viewer Zero Footprint Client 6.0 software from GE HealthCare (Chicago, USA), dedicated to the analysis of DICOM images (open-source software; https://www.gehealthcare.it/).

**Fig. 2:**
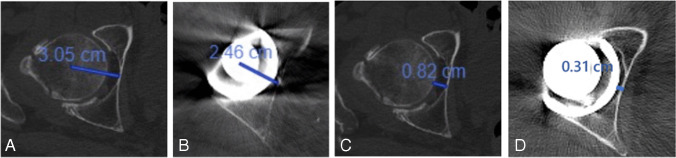
TC evaluation of the pre-operative AO (**A**), post-operative AO (**B**), pre-operative AFd (**C**), and post-operative AFd (**D**)

All the measurements were carried out on the transverse plane in the section running through the level of the true floor of the acetabulum and in the middle of the femoral head (i.e., at the level of its greatest diameter). AO was defined as the distance between the acetabular floor (i.e., the inner wall of the quadrilateral plate) and the centre of the femoral head. The Acetabular Floor distance (AFd) was defined as the distance between the most medial point of the femoral head and the acetabular floor (Fig. 2). Acetabular version (AV) was measured on the transverse plane running through the middle of the femoral head at the level of its greatest diameter. A first line connecting the anterior and posterior wedges of the native acetabulum (or the acetabular cup, post-operatively) and a second line connecting the ischiatic spine were drawn. AV was defined as the complementary angle to the angle between these two lines. The same measurements were carried out at six months post-operatively.

All the patients received an uncemented THA through a direct lateral approach. Patients were positioned in a full lateral position on a standard orthopaedic table. A straight lateral skin incision centered on the greater trochanter was performed, starting 3–5 cm proximal to the trochanteric tip and extending distally for 5–10 cm. Fascia lata was identified and incised in line with the skin incision and retracted with a self-retaining retractor. The trochanteric bursa was incised to demonstrate the anterior and posterior borders of the gluteus medius and vastus lateralis muscle. The gluteus medius was incised at the myotendinous junction on the greater trochanter. The inferior branch of the superior gluteal nerve was protected by not extending the gluteus medius detachment proximally. The insertion of the vastus lateralis muscle was preserved. An anterolateral capsulectomy was performed, and the hip was dislocated by flexion, adduction and external rotation. The operative leg was placed into a sterile pouch to perform femoral neck osteotomy. The acetabular reaming technique varies in the two groups. In group A, the peripheral acetabular reaming technique was performed through a single-use sterile reamer (Groupe Lépine™, Genay, France). The removed femoral head was measured, and a single-use sterile reamer 2 mm higher than the native femoral head was open and used to obtain a peripheral and more anatomical preparation of the acetabulum. The acetabular cup (Quattro™ Hap Cup VPS PnP, Groupe Lépine™) was placed such that its version likely reproduced the native one and limiting the overhang respect to the acetabular rim to reduce the risk of soft tissue or bony impingement. In group B, a “conventional” technique was performed and consisted in reaming the acetabulum by sequentially larger reamers until cancellous bone in the acetabular floor and equatorial zone was exposed. The acetabular cup (Trinity™ acetabular cluster shells, Corin Group, Cirencester, GL7 1YJ, UK) was lying in the exposed acetabular floor, and a medialisation of the hip COR occurred.

The radiological evaluation was performed by the same observer (V.G.) blinded to the surgical technique used.

Statistical analysis was performed using SPSS 19.0 software (IBM). In each group, pre- and post-operative differences and differences between the two groups were analyzed. The Student *t* test was used to compare the two groups. The two groups were compared with respect to patient demographic data. Statistical significance was defined as *p* < 0.05.

## Results

The demographic data were similar in both groups (Table 1). No cases of intra-operative or post-operative acetabular component malpositioning/mobilization occurred. No cases of peri-acetabular fracture, early-onset infection, or dislocation occurred.

**Table 1:** Demographic data

	Single-use reaming	Conventional reaming
Patients	40	40
Sex	14 M, 26F	14 M, 26F
Age	69 yy	71 yy
BMI	31	30
Side	19 R, 21 L	23 R, 17 L
Approach	Direct lateral	Direct lateral

In group A, the mean pre-operative AO was 2.89 cm and post-operatively changed to 2.47 cm (mean difference 0.42 cm). In group B, the mean pre-operative AO was 3.31 cm and post-operatively changed to 2.38 cm (mean difference 0.93 cm). The mean pre- to post-operative difference between group A and group B was statistically significant (*p* value < 0.05).

In group A, the mean pre-operative AFd was 0.67 cm and post-operatively changed to 0.31 cm (mean difference 0.36 cm). In group B, the mean pre-operative AFd was 0.91 cm and post-operatively changed to 0.25 cm (mean difference 0.66 cm). The mean pre- to post-operative difference between group A and group B was statistically significant (*p* value < 0.05).

The mean anteversion was 17° ± 3° and 15° ± 4° in group A and group B, respectively. The difference was not statistically significant. There were cases of anterior overhang and two cases of posterior overhang in group A, which were not related to any impingement symptoms or clinical complains by patients (Table 2).

**Table 2:** Comparison between groups for pre- and post-operative AO and AFd difference (Δ), anteversion and surgical time

	Study group			Control group			*p* value
	PRE	POST	Δ	PRE	POST	Δ	
AO (cm)	2.89	2.47	0.42	3.31	2.38	0.93	*p* < 0.05
AF (cm)	0.67	0.31	0.36	0.91	0.25	0.66	*p* < 0.05
Anteversion	17° ± 3°			15° ± 4°			*p* > 0.05
Surgical time	48 ± 7 min			54 ± 9 min			*p* < 0.05

The mean surgical time was 48 ± 7 min and 54 ± 9 min in group A and B, respectively. The difference was statistically significant (*p* value < 0.05).

## Discussion

The main finding of the current prospective study is that the “single-use peripheral” reaming technique demonstrated to be more reliable in reproducing the native COR and AO of patients undergoing to primary THA than the “conventional” one. Thus, the hypothesis has been confirmed. Furthermore, a statistically significant lower surgical time was found with the “single-use peripheral” reaming technique, even though it was not associated with a reduction of the risk of infection.

Nowadays, the importance of restoring offset and COR of native hip in THA has been ascertained by orthopaedic surgeons [1–5, 24, 25]. According to the CT-based study of Seriali et al. [12], the mean FO was 42.2 ± 5.1 mm, which is not much higher than the mean AO found in the current (33.1 mm) or other similar studies [26]. AO has been only recently considered as a determining factor in respecting native COR [26, 27]. It has been suggested that COR should not be displayed more than 3 mm superiorly and 5 mm medially, and the impact of different reaming technique in modifying AO and hip COR has been recently highlighted [10]. Comparing conventional and peripheral reaming techniques, Bonin et al. [26] reported a much higher COR displacement with conventional reaming (i.e., COR displacement of 5 mm medially and 3.7 mm superiorly with conventional reaming vs 0.8 mm and 0.7 mm with peripheral one). For this reason and because acetabular anatomy widely varies between humans, surgeons need to understand well the different acetabular reaming techniques to guarantee proper hip function and longevity.

According to the result of the current study, a peripheral reaming technique through a “single-use sterile” instrumentation allows for a more reliable restoration of the AO and COR. Peripheral reaming, as previously reported [8], may provide an improvement in ROM and abductor muscle lever arm, and at the same time, a reduction in the risk of impingement (both bone or soft tissue related) and consequently dislocation [8, 19, 20, 23]. Garcìa-Rey and Garcìa-Cimbrelo [19] found that after THA, the risk of dislocations is higher if the acetabular cup has a greater acetabular abduction angle or a greater medialisation of the COR in the post-operative radiographs. Kurtz et al. [8] demonstrated that AO changing has a greater effect than FO changing on ROM of the hip after THA. Furthermore, peripheral reaming preserves the acetabular bone stock which could be beneficial in case of implant failure and revision surgery (Jeffrey J Raj et al.) [28].

Lastly, the use of a “single-use sterile” reamer as presented in the current study may provide additional advantages in terms of infection risk and operative time. Many studies in literature evaluated the association between operative time and the infection risk in THA [29]. Wang et al. [30] found that a 20-min increase in operative time was associated with up to 25% increased of periprosthetic joint infection (PJI). Scigliano et al. [31] in their systematic review reported that the risk of PJI in primary joint replacement is significantly higher if the operative time is greater than 120 min. In the current study, a reduction of six min in the operative time was found. The reduction was statistically significant. Therefore, using a “single-use sterile” reamer may lead to a reduction in operative time and subsequential infection risk which should be taken into account by surgeons.

On the other hand, the peripheral anatomical technique also presents some pitfall that need to be mentioned. Firstly, the acetabular cup may be superolaterally uncovered. In this scenario, surgeons should keep in mind that if by searching an “in-line position” with the acetabular superior rim, a risk of a vertical placement of the acetabular cup may occur [26, 32, 33]. Secondly, if the acetabular cup is not anteverted enough, anterior overhang and iliopsoas impingement can occur [34]. Conversely, if an excessive anteversion is pursued, a posterior overhang can occur. Regarding wear and long-term loosening, discordant results have been reported in literature, and no clear conclusion can be drawn. Enrico De Pieri et al. [35], in their study on virtual musculoskeletal simulations, compared medialised versus anatomical reconstruction in THA and reported that cup medialisation provides just small biomechanical advantages, and a fully medialised reconstruction is not recommended.

In order to preserve the hip COR other authors [36–39], suggested to use an extended offset polyethylene acetabular liner to move the COR away from the plane of the acetabular metallic shell with an improvement in soft tissue balancing and stability. However, it may increase torsional forces at the liner-shell and bone-implant interface with a subsequent increase in the risk of implant wear and acetabular aseptic loosening as reported by Archibeck MJ [37].

To the best of our knowledge, this is the first study which reported results of a “single-use peripheral” acetabular reaming technique in reproducing COR and AO in primary THA. To the best our knowledge, this is also the first in vivo study comparing through a CT scan conventional and peripheral acetabular reaming in such series of patients. These two represent the main strengths of the study. Furthermore, all patients were treated by the same surgeon, and the inclusion and exclusion criteria together with the prospective nature of the study contribute to increase the strength of the results, because various confounding factors could be likely eliminated.

The current study also presents some limitations which needed to be mentioned. Firstly, an evaluation of clinical outcomes was not performed, and it is not possible to ascertain if the anatomical technique provides better clinical results. Of course, it could be an interesting and useful point to be investigated, but it is beyond the aim of the current study. Secondly, a single expert radiologist evaluated all the CT scans, and no intra-/inter-observer correlation was performed. However, the evaluated radiological parameters are quite simple and not categorical, and this could likely mitigate the limitation. Lastly, two different cups were used in the two groups. However, this could not be such a great limitation, because it is more the reaming technique to condition the cup position rather than the industry producing the cup itself.

## Data Availability

Data sets generated during the current study are available from the corresponding author on reasonable request.

## Declarations

All authors contributed to the study conception and design and all authors read and approved the final manuscript. Informed consent was obtained from all individual participants included in the study.
