# Correction to: “Single-use peripheral” vs “conventional” reaming in total hip arthroplasty: how to respect native centre of rotation and acetabular offset? A CT study

**DOI:** 10.1007/s00264-023-05986-5

**Published:** 2023-09-15

**Authors:** Edoardo Viglietta, Leonardo Previ, Veronica Giuliani, Giulia Rescigno, Yuri Gugliotta, Andrea Redler, Raffaele Iorio

**Affiliations:** Orthopaedic Unit, S. Andrea Hospital, University of Rome, “La Sapienza” Via Di Grottarossa 1035, Rome, Italy

**Correction to: International Orthopaedics**

10.1007/s00264-023-05899-3

All the author names are inverted in the original published paper, the correct names are given below:

- Edoardo Viglietta
  Leonardo Previ
  Veronica Giuliani
  Giulia Rescigno
  Yuri Gugliotta
  Andrea Redler
  Raffaele Iorio

The original article has been corrected.

